# Supplementary material for: Robust ultraclean atomically thin membranes for atomic-resolution electron microscopy
Source: Nat Commun. 2020 Jan 28;11:541. doi: 10.1038/s41467-020-14359-0 (PMC6987160; doi:10.1038/s41467-020-14359-0)
Supplement: Supplementary file 1 — Supplementary Information [file 41467_2020_14359_MOESM1_ESM.pdf]

Supplementary Information for

Robust ultraclean atomically thin membranes for atomic-resolution electron microscopy

Liming Zheng *et al.*

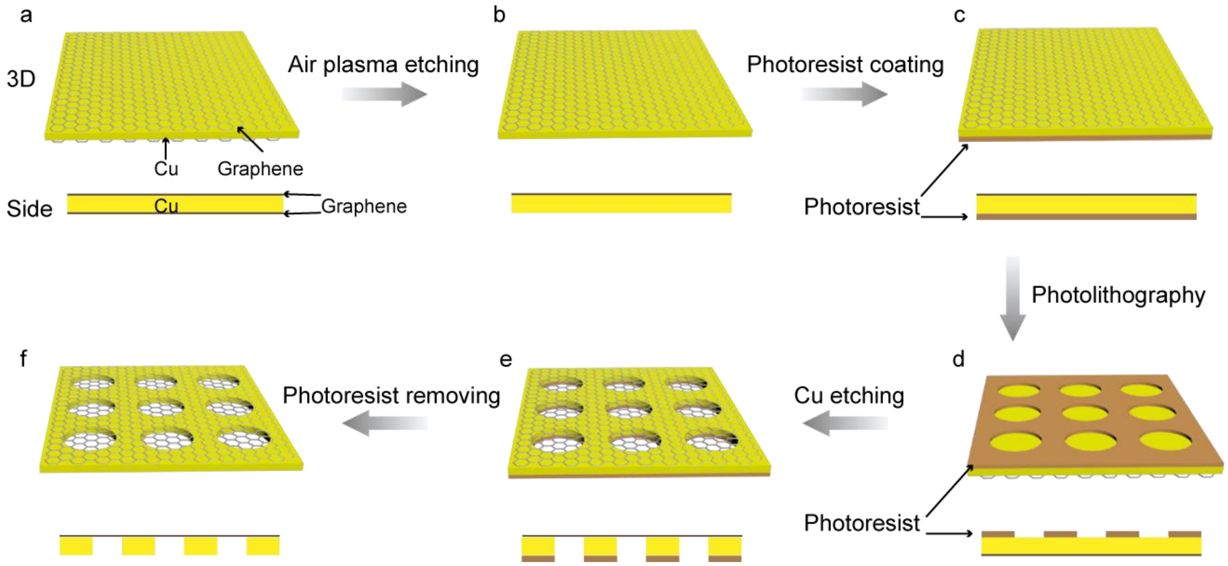

Supplementary Figure 1. Fabrication process of the graphene membranes. a, CVD-grown graphene films on both sides of Cu foil. **b,** Removal of the graphene from one side of the Cu by air plasma etching. **c,** Coating the Cu side in **b** with photoresist. **d,** Patterning the photoresist by the conventional photolithography. **e**, Selective etching the Cu substrates with the $Na_2S_2O_8$ aqueous solution. **f**, Removing the photoresist. After rinsing and drying, the graphene membranes are successfully fabricated.

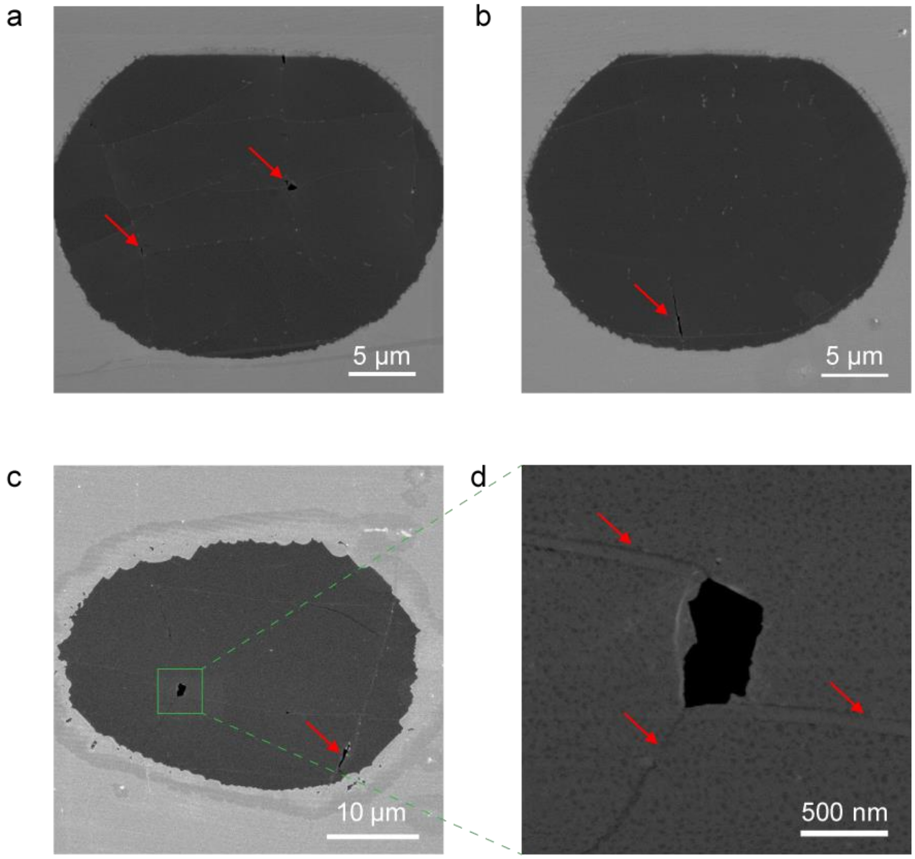

Supplementary Figure 2. Mechanical failure of suspended graphene in the linear defects after drying. a-b, SEM images showing the cracks along the linear defects of suspended graphene. The red arrows reveal the linear defects. **c-d,** SEM images show the broken suspended graphene at the junction of linear defects.

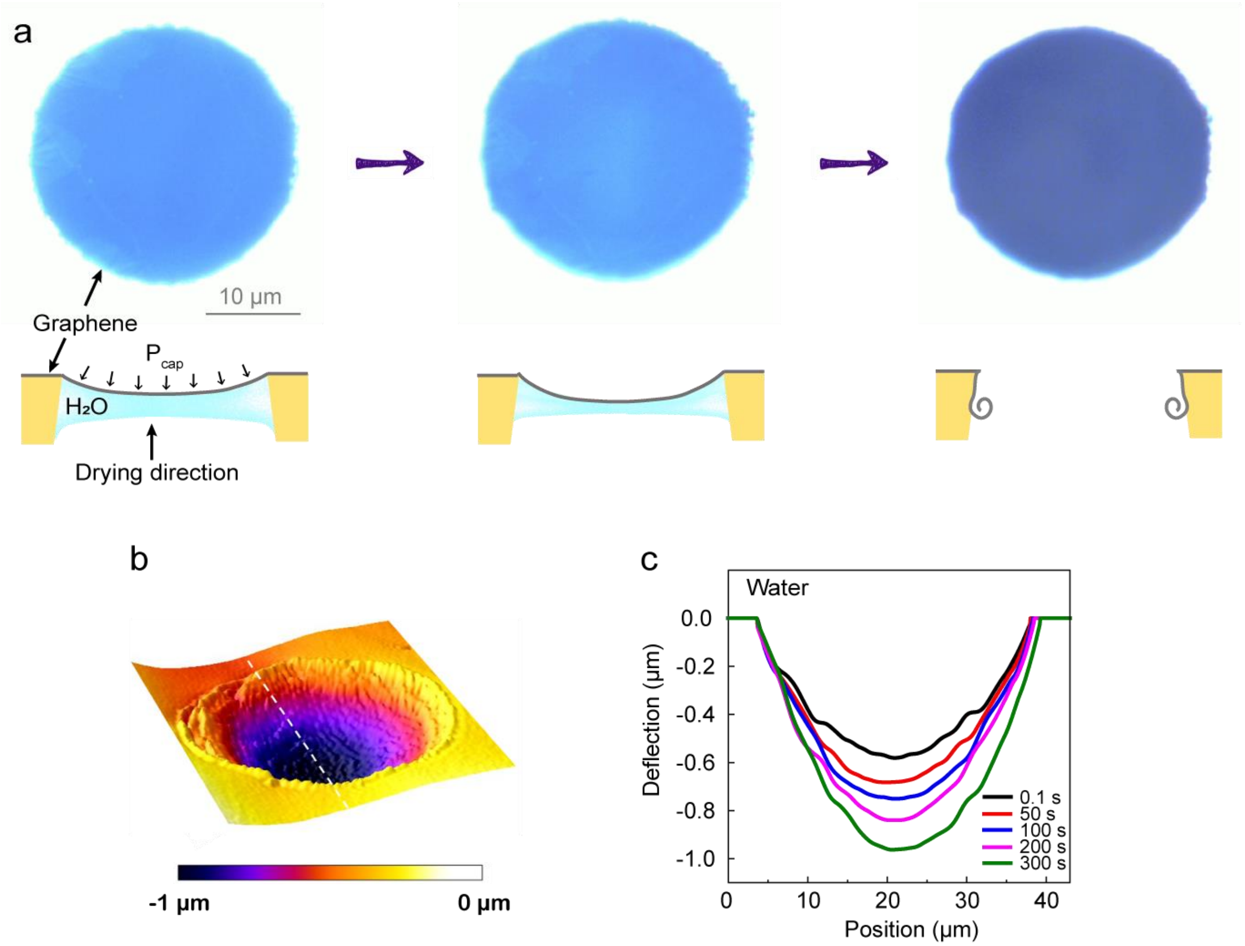

Supplementary Figure 3. Downward deformation of graphene membrane due to the capillary pressure during the liquid volatilization. a, Optical images and corresponding schematic illustrations reveal the deformation behaviors of graphene under the capillary pressure during the drying process, and the graphene membrane was broken and pulled down by water. **b,** Representative topographic image of the deformed graphene under the capillary pressure taken by the digital holographic microscope. **c,** Deflection profile history obtained from the graphene membrane in **b** along the white dash line. The deflection of graphene membrane increases gradually during the water evaporation. Note that, the “0.1 s” was the time we started recording images.

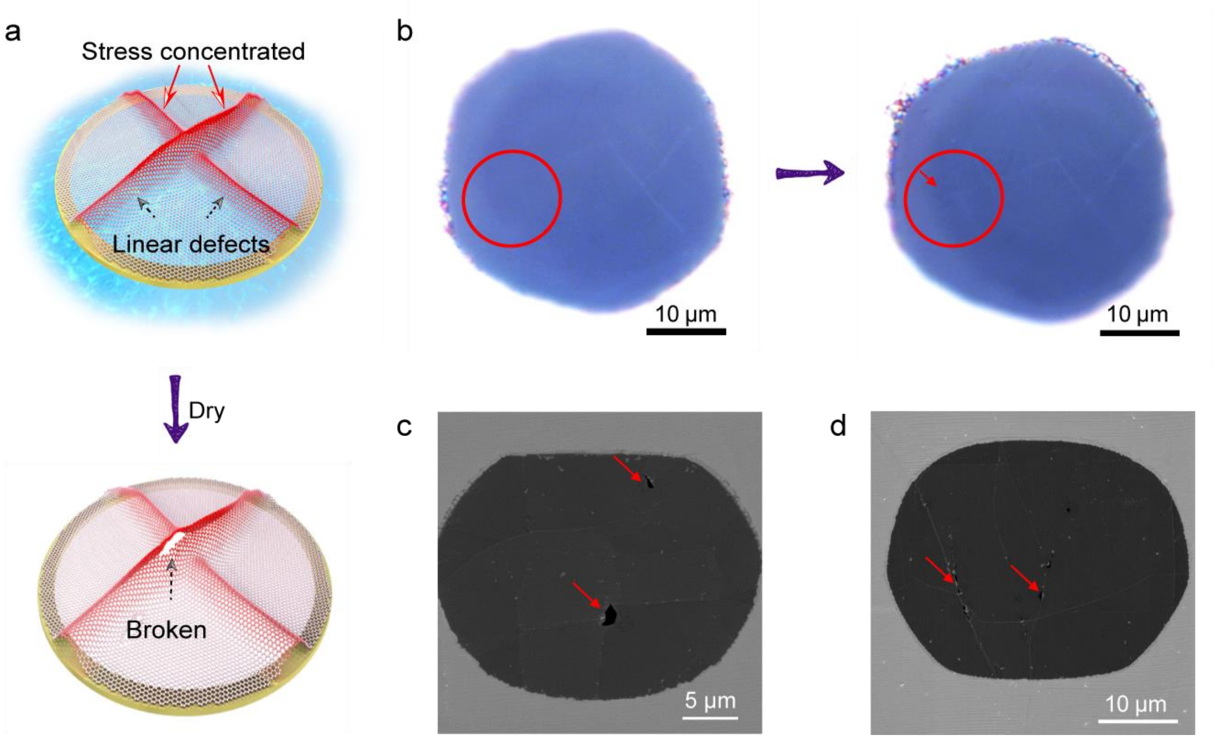

Supplementary Figure 4. Cracks formation in the linear defects of graphene membrane during the drying process. a, Schematic illustration of graphene mechanical failure in the linear defects, especially at the junction. **b,** *In-situ* optical observations of the drying process. The crack appeared in the linear defects of graphene membranes. The red arrow reveals the crack. **c-d,** SEM images showing the cracks at the junction (**c**) and along the linear defects (**d**). When the liquid was not dried totally, the graphene membranes were processed by critical-point drying, which can eliminate the surface tension of the liquid to prevent the graphene membranes with cracks from totally ruptured. In this way, the cracks formed during the drying process can be captured.

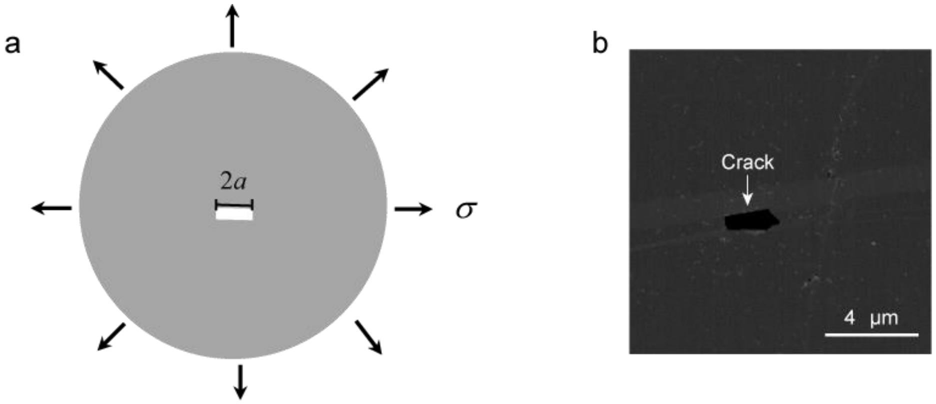

Supplementary Figure 5. The effects of cracks on mechanical properties of suspended graphene membranes. A cracked graphene under pressure can be simplified to an axisymmetric model as shown in (**a**). The stress intensity factor K_I near the crack tip can be expressed as

$$K_I = \sigma(\pi a)^{1/2}.$$

Here, σ is the strength of a cracked suspended graphene, a is the characteristic length of the crack in suspended graphene, which is estimated to be 0.1~2 μm as shown in (**b**). And the reported inherent fracture toughness of pristine graphene[1] K_{Ic} is 4±0.6 MPa·m$^{1/2}$.Considering that $K_I = K_{Ic}$, we can get the following equation.

$$\sigma = K_{Ic}(\pi a)^{-1/2}$$

Then the strength of a cracked graphene σ will be reduced to 1~10 GPa.

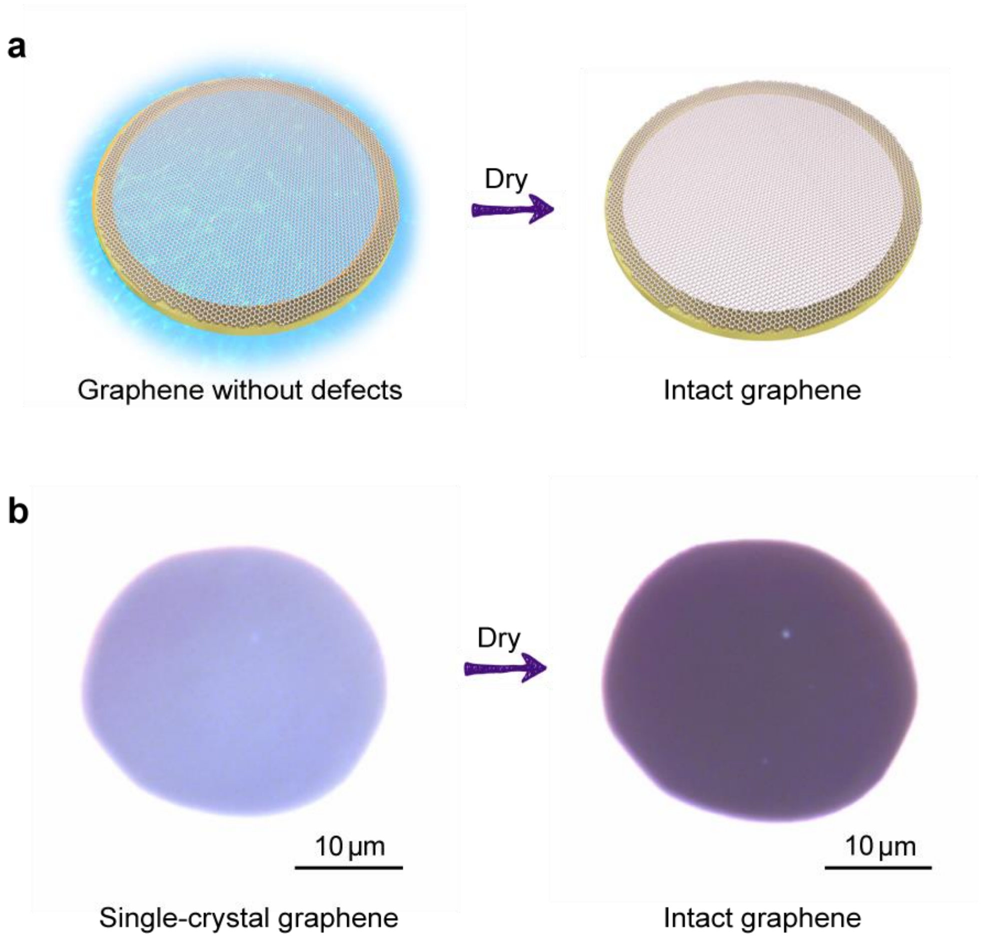

Supplementary Figure 6. Intact suspended graphene membrane without linear defects after drying. a, Schematic illustration showing the graphene membranes keep intact after the liquid evaporation. **b,** *In-situ* optical observations of the drying process. The single-crystal graphene membrane without wrinkle was robust enough to withstand the capillary pressure and the surface tension of the liquid. Note that, the white particles loaded on the graphene membrane reveal the existence of graphene membrane.

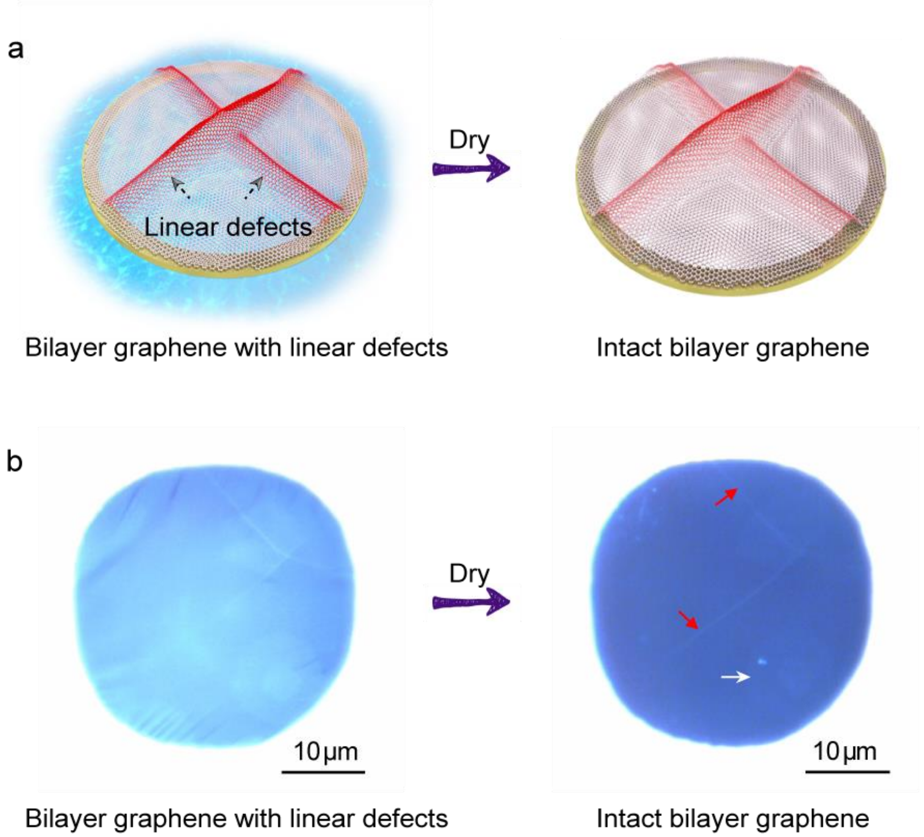

Supplementary Figure 7. Intact bilayer graphene membrane during the drying process. a, Schematic illustration shows the bilayer graphene membranes with linear defects keep intact after drying. **b,** *In-situ* optical observations showing the bilayer graphene membrane remains intact after the liquid totally evaporated. The linear defects and small multilayer island were clearly distinguished on the bilayer graphene.

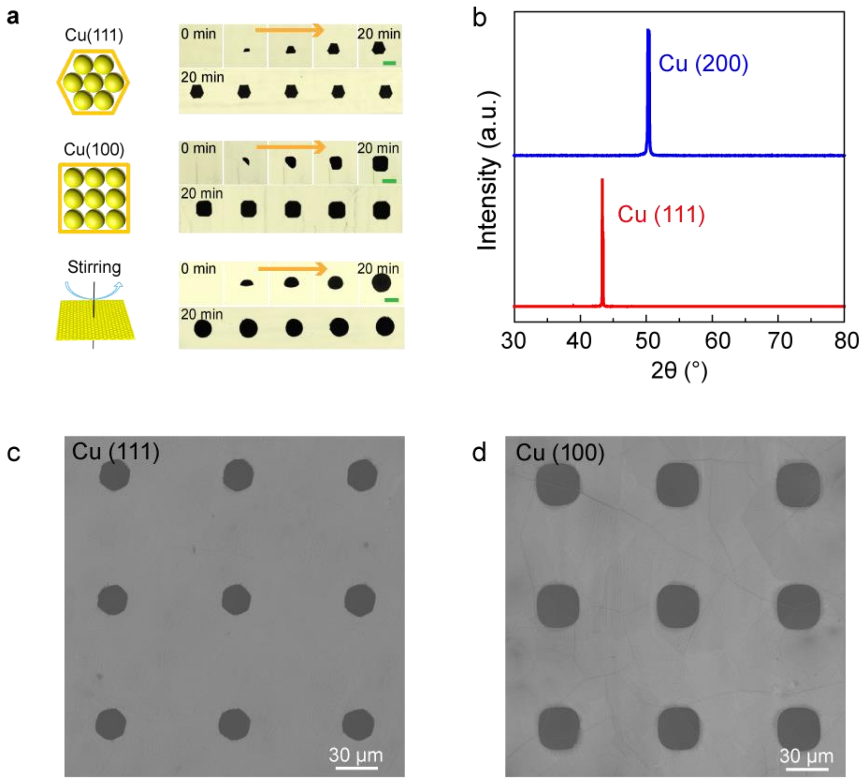

Supplementary Figure 8. Morphological evolution and structural control of graphene membranes. a, The shape control of the suspended graphene membranes. The *in-situ* optical observations presenting the formations of the hexagonal shape on Cu(111) foils, square shape on Cu(100) foils and circular shape under stirring operations. Scare bar, 30 μm. **b,** XRD patterns of Cu(111) and Cu(200). **c,** SEM image of hexagonal suspended graphene membranes on Cu(111) foil. **d,** SEM image of square suspended graphene membranes on Cu(100) foil.

We investigated the microstructures and surface properties of the graphene membranes. *In situ* optical microscopy images during the etching process of graphene/Cu foil showed the morphological evolution and structural control of graphene membranes suspended on Cu grids (Supplementary Fig. 8a). The etching behavior on the Cu (111) and the Cu (100) substrates was thermodynamically anisotropic without an operation of stirring during the etching process, resulting in hexagonal and square shapes of the hole with suspended graphene membranes, respectively (Supplementary Figs. 8b-8d). In contrast, a continuous stirring of the etching reaction solution could result in the formation of round-shaped holes with suspended graphene membranes on Cu foils despite the orientation of the Cu lattice. Considering that the round-shaped holes might suppress the stress on the suspended graphene membranes and maximize the field of view in EM, we mostly used these types of graphene membranes for further studies.

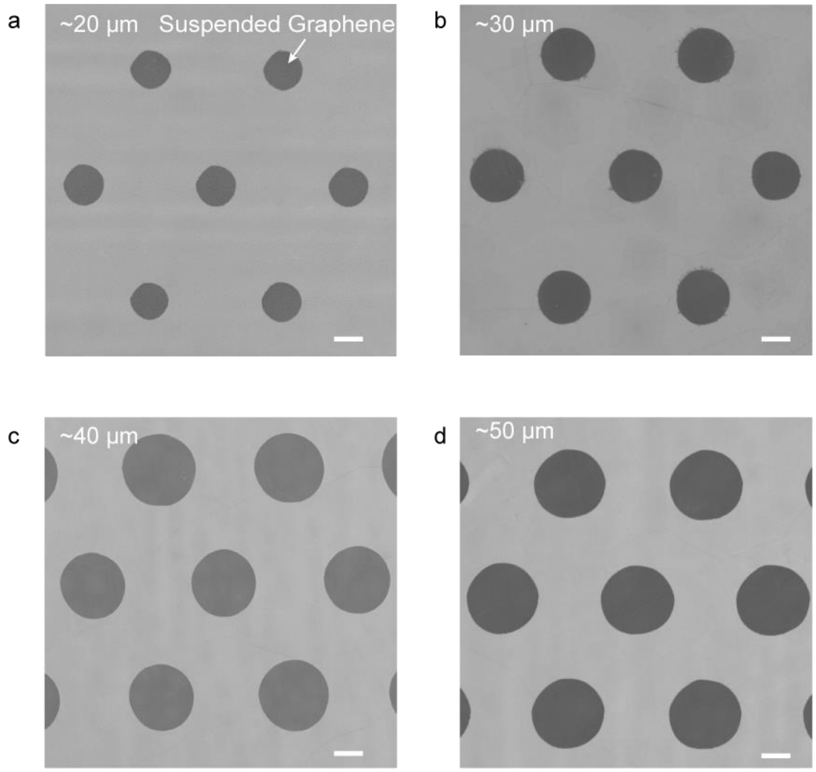

Supplementary Figure 9. Size control of graphene membranes. a-d, SEM images showing the suspended graphene membranes with a lateral size of ~20 μm (**a**), ~30 μm (**b**), ~40 μm (**c**) and ~50 μm (**d**), respectively. Scare bars, 20 μm.

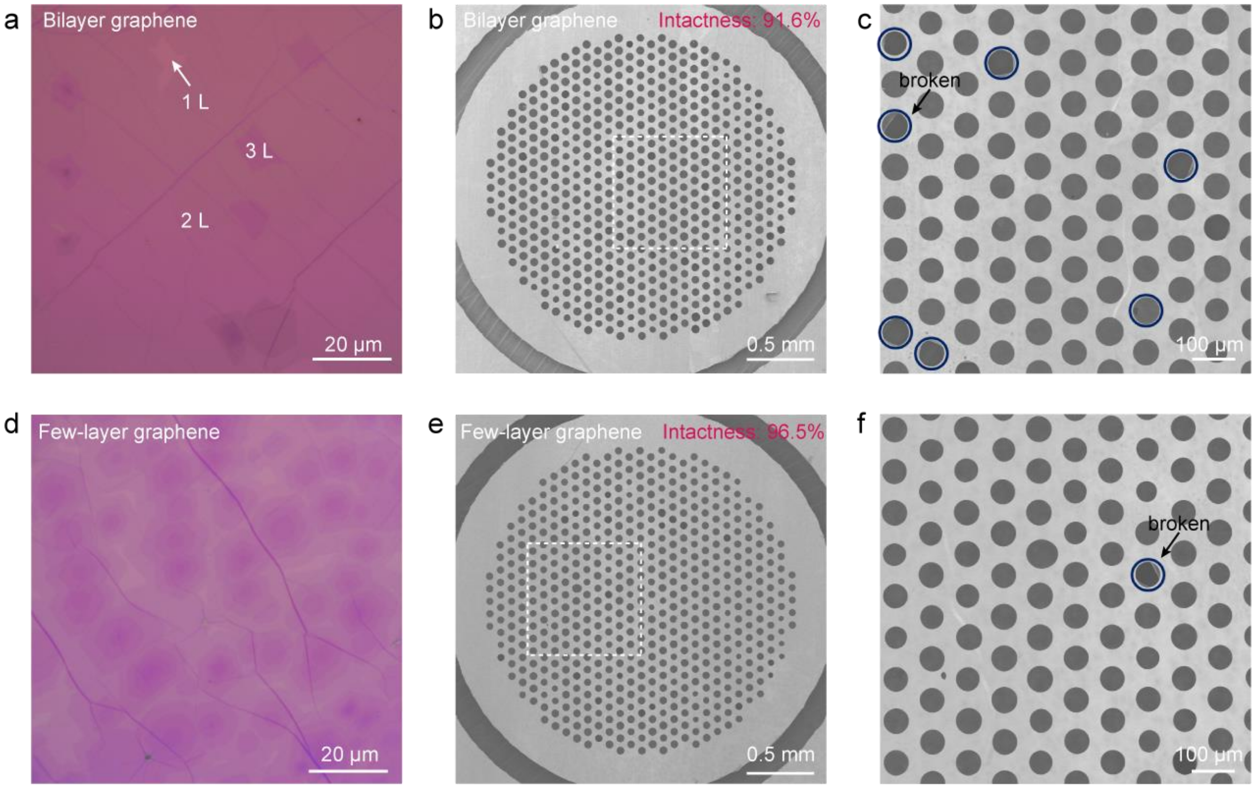

Supplementary Figure 10. Characterizations of bilayer and few-layer graphene membranes. **a,** Optical microscope image of bilayer graphene film transferred onto SiO_2/Si substrate. **b,** SEM image of the bilayer graphene grid with an intactness of 91.6%. **c,** Suspended bilayer graphene membranes from the marked region in **a**, the holes in the cycles are the regions uncovered by graphene or covered by broken graphene films. **d,** Optical microscope image of few-layer graphene film transferred onto SiO_2/Si substrate. **e,** One typical few-layer graphene grid with an intactness of 96.5%. **f,** SEM image of the few-layer suspended bilayer graphene membranes.

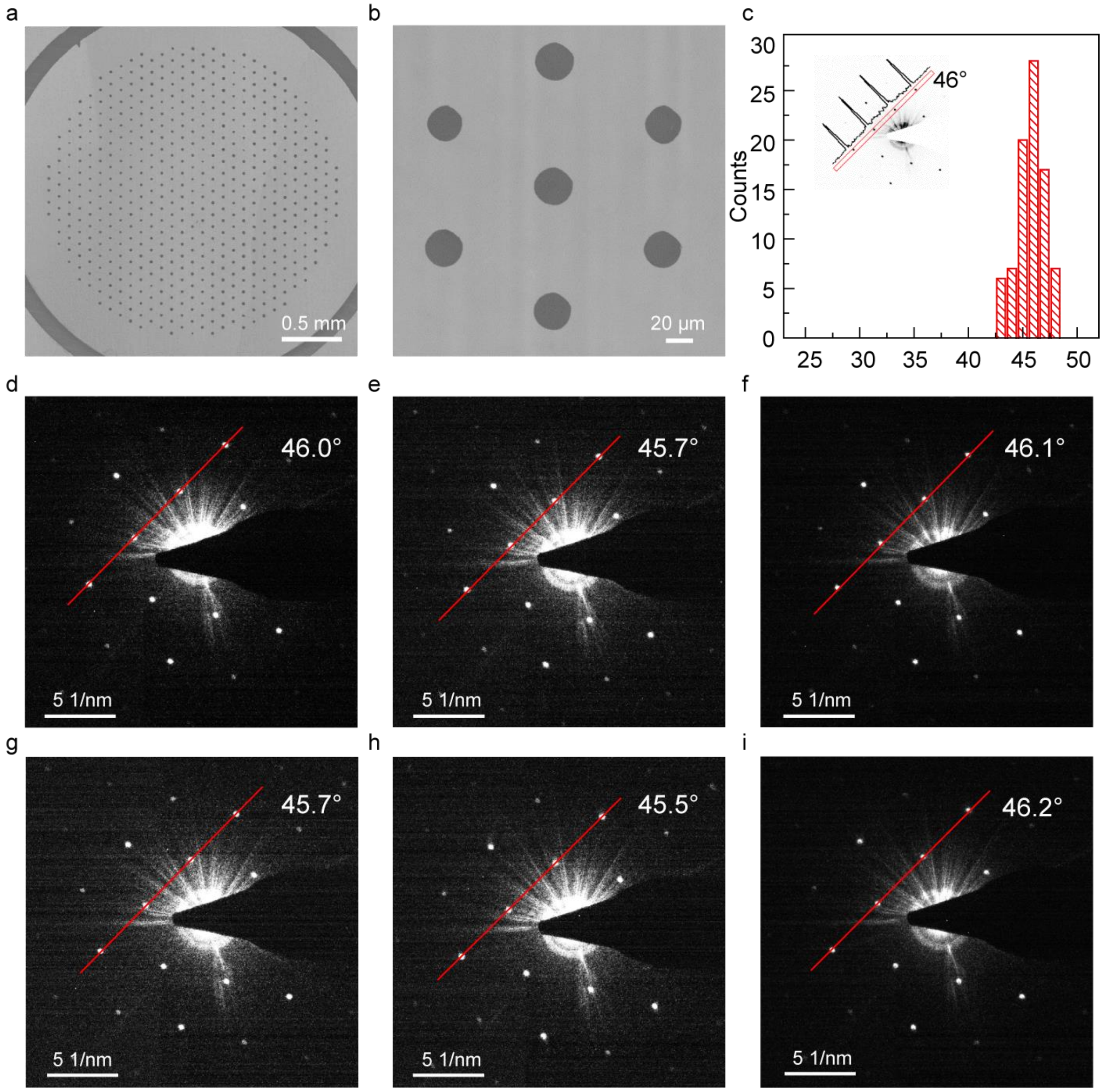

Supplementary Figure 11. Characterizations of single-crystal graphene membranes. a, SEM image of one single-crystal graphene grid with an intactness of 90%. **b,** SEM image of suspended graphene membranes from the graphene grid in **a**. **c,** Angle distribution from extensive selected area electron diffraction (SAED) patterns within the graphene grid in **a**, the narrow distribution of angle at 45.7 ± 2.5° indicates that the graphene is single-crystal. Inset: representative SAED pattern and the intensity profile of the diffraction pattern in the red box. **d-i,** Representative selected area electron diffraction (SAED) patterns from one single-crystal graphene grid. Source data are provided as a Source Data file.

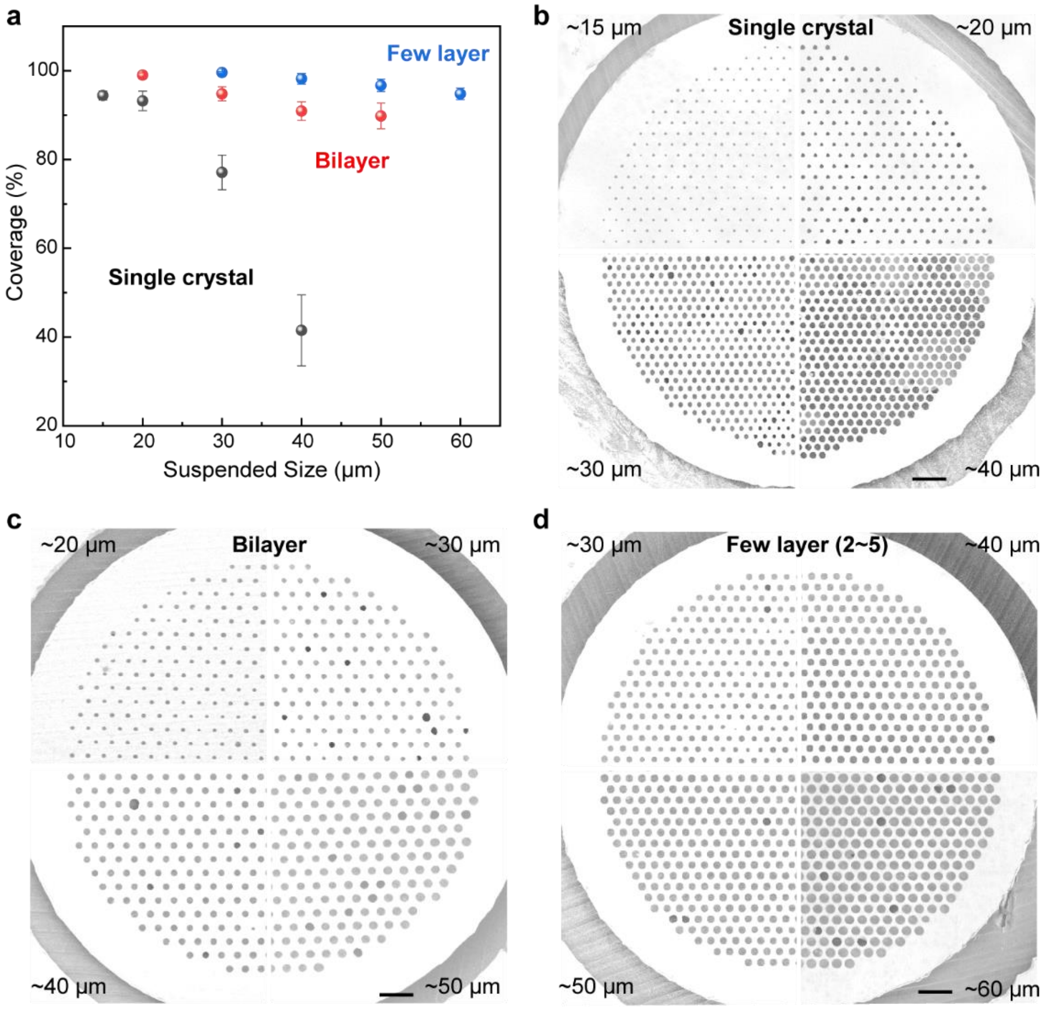

Supplementary Figure 12. Coverage of suspended graphene membranes with different diameters. a, Statistical graph showing the coverage of single-crystal, bilayer and few-layer suspended graphene membranes with different suspended sizes. **b,** SEM images of single-crystal suspended graphene membranes with the diameter of ~15 μm, ~20 μm, ~30 μm and ~40 μm, respectively. **c,** SEM images showing the bilayer suspended graphene membranes with the diameter of ~20 μm, ~30 μm, ~40 μm and ~50 μm, respectively. **d,** SEM images of few-layer suspended graphene membranes with the diameter of ~30 μm, ~40 μm, ~50 μm and ~60 μm, respectively. Source data are provided as a Source Data file.

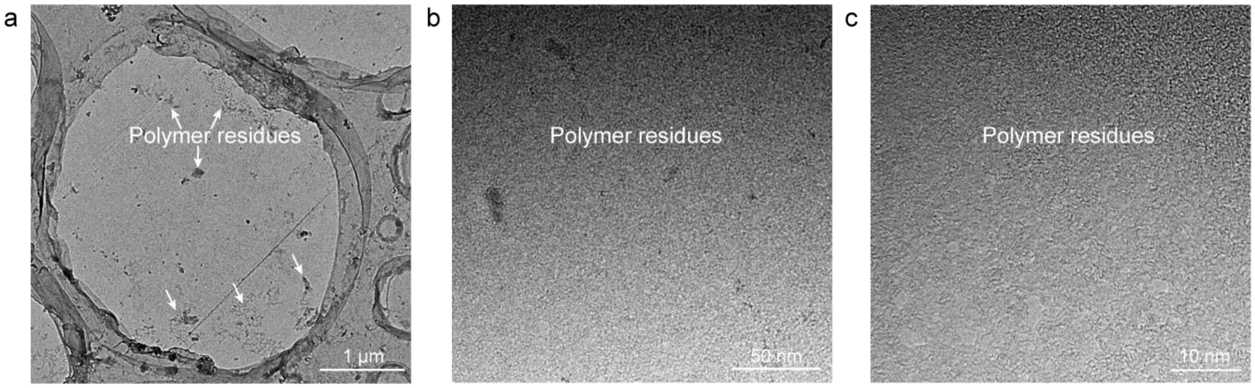

Supplementary Figure 13. TEM images of suspended graphene membrane fabricated by polymer-film-mediated transfer technique. a, Typical low-magnification TEM image of suspended graphene on a 3 μm hole, where the white arrows indicate the abundant polymer residues. **b-c,** Representative high-magnification TEM images of suspended graphene showing the graphene surface is almost completely covered by the polymer residues.

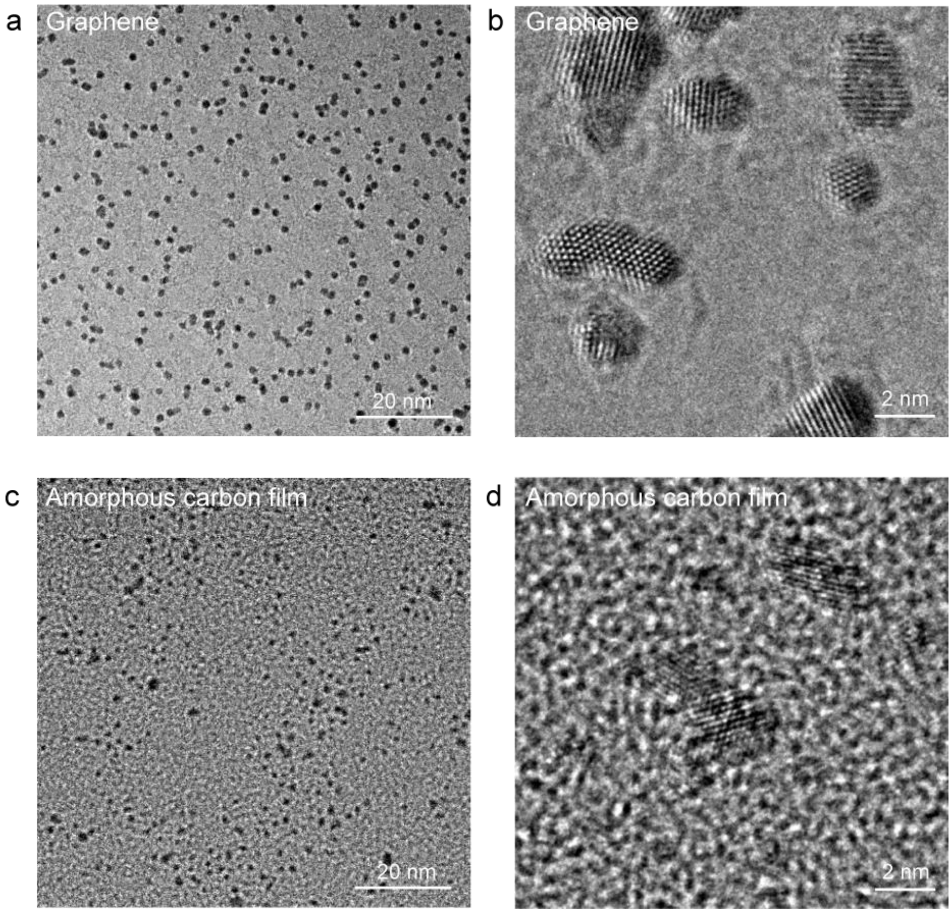

Supplementary Figure 14. Improved TEM images of Pt particles on the graphene membranes. a, TEM image showing the uniform distribution of Pt nanoparticles on the graphene. **b,** High-magnification TEM image of Pt nanoparticles, where the lattice fringes of Pt particles were clearly observed. **c-d,** TEM images of Pt nanoparticles on the amorphous carbon film. Graphene can improve the quality of TEM images compared to the amorphous carbon film which induces the extra chromatic aberration.

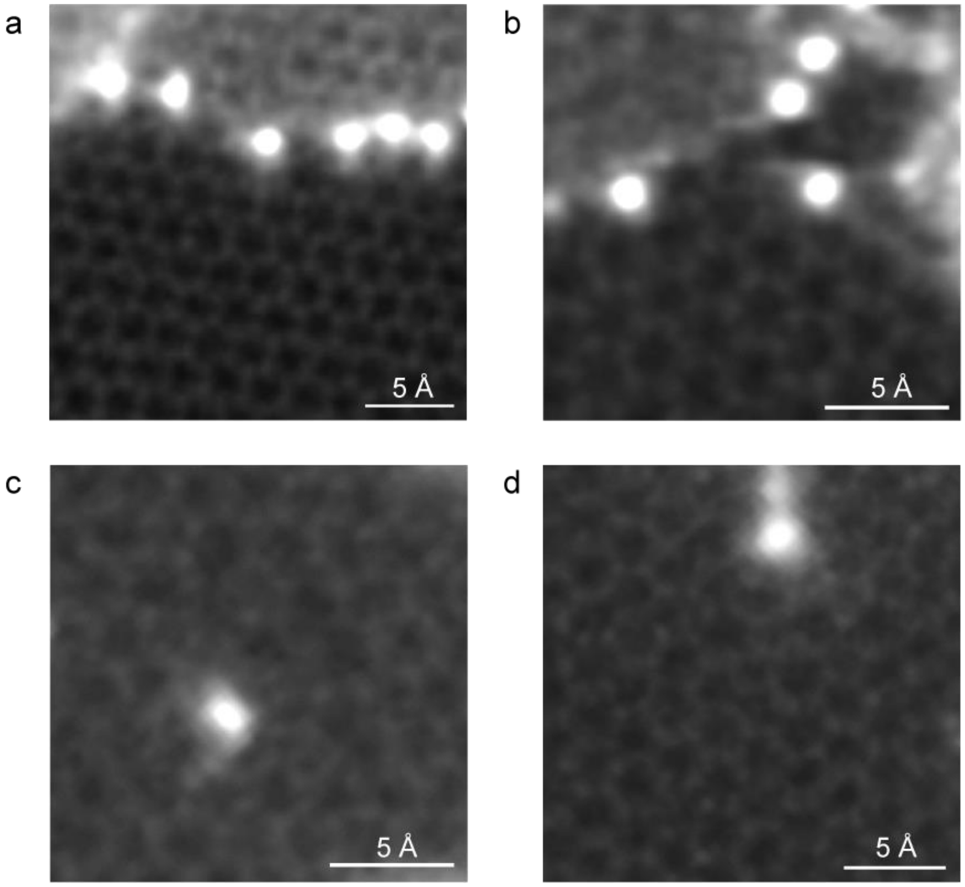

Supplementary Figure 15. HAADF-STEM images of the Cu single atoms loaded on the graphene membranes. a-d, Individual Cu atoms are clearly observed on the graphene. The brighter hexagonal lattices in the upper region of **a** and **b** are the atomically thin graphitized carbon adsorbates.

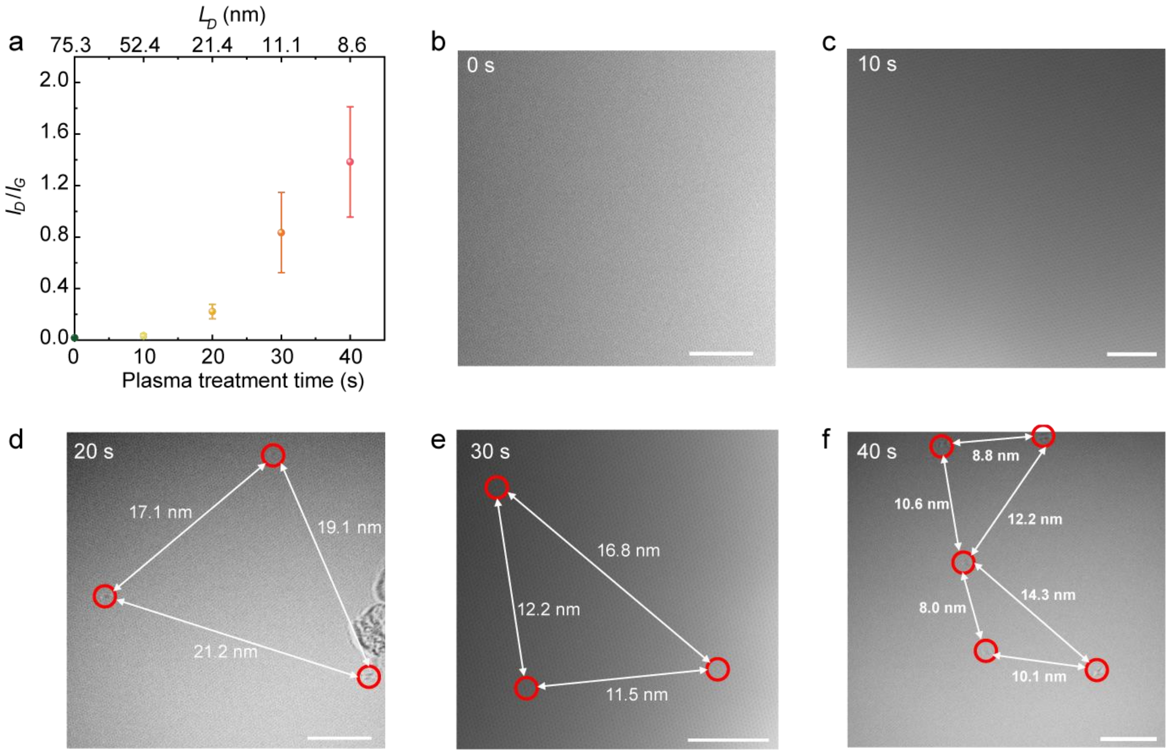

Supplementary Figure 16. Quantifying the site-defect density in plasma-treated graphene. a, The variable intensity ratios of the D and G peaks (I_D / I_G) with corresponding average distance between defects (L_D). Not that L_D is plotted as top x axis, and L_D is showed at the corresponding oxygen plasma treatment time without any linear relationship. The values of oxygen-containing active sites density σ from 0 s to 40 s are calculated to be 1.76×10^{-4} nm^{-2}, 3.64×10^{-4} nm^{-2}, 2.18×10^{-3} nm^{-2}, 8.12×10^{-3} nm^{-2} and 1.35×10^{-2} nm^{-2} using $\sigma=1/L_D^2$, respectively. **b-f,** High-resolution TEM images of defects in the graphene with different plasma treatment time: 0 s (**b**), 10 s (**c**), 20 s (**d**), 30 s (**e**), 40 s (**f**). The black dots in red circles reveal the point defects that correspond to the oxygen adducts[2]; and the distances between defects are marked with the white arrows. Scale bars, 5 nm. Source data are provided as a Source Data file.

Raman spectroscopy is powerful in quantifying the defects in graphene.[3,4] Typically, the defect density σ can be described by the average distance between defects L_D, where $\sigma=1/L_D^2$. And L_D can be calculated from the intensity ratio of the D and G bands (I_D/I_G) in Raman spectra.[5] In the low defect density regime (L_D >6 nm), I_D/I_G = C/L_D^2; and in the high defect density regime, I_D/I_G = D·L_D^2. For the oxygen-plasma induced defects in graphene, the C is reported to be 102 nm^2 and D is obtained by imposing continuity between the two regions.[2,5,6] In our cases, the maximum I_D/I_G

is ~1.8 (Figure 3b) and the corresponding L_D is ~8 nm (L_D >6 nm), revealing the low defect density in the functional graphene. Thus, we can calculate the average distance between defects (L_D) using $I_D/I_G = 102/L_D^2$.

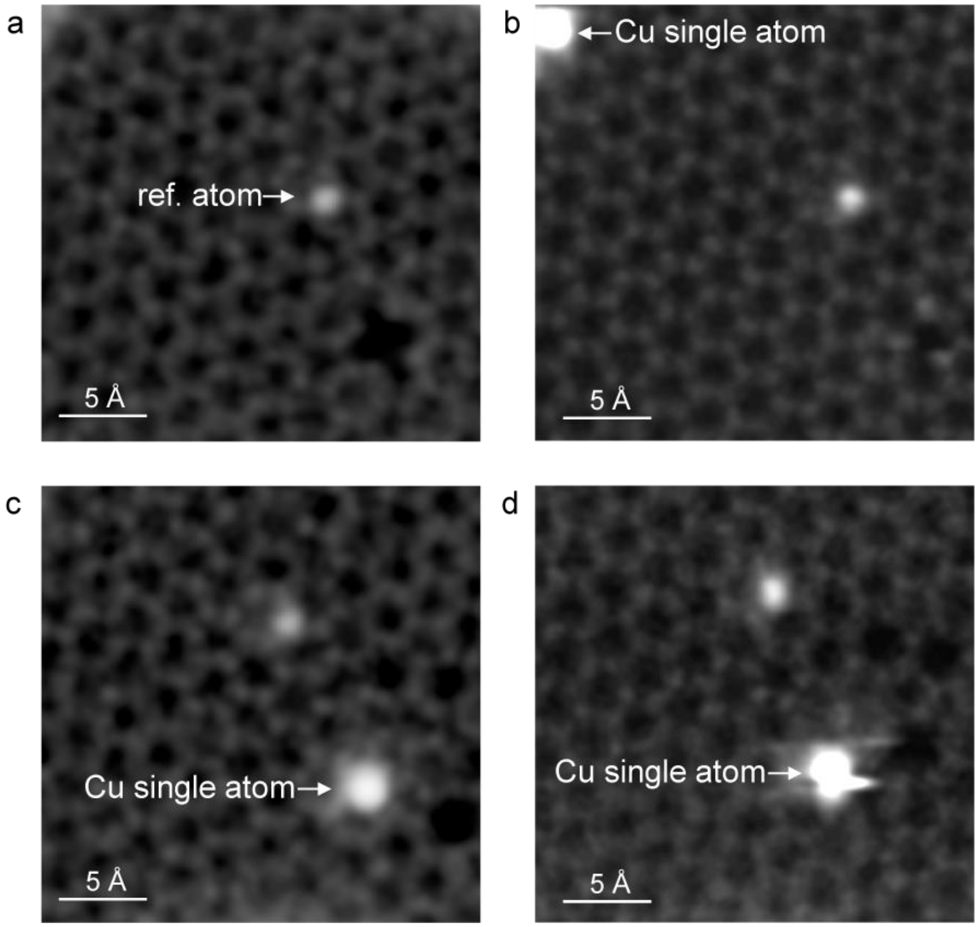

Supplementary Figure 17. Stabilization of Cu single atom in the graphene lattice defect. a-d, HADDF-STEM images revealing the movement of Cu single atom to the active site under the electron beam radiation. Note that the reference atom in the graphene lattice was Si single atom and kept still.

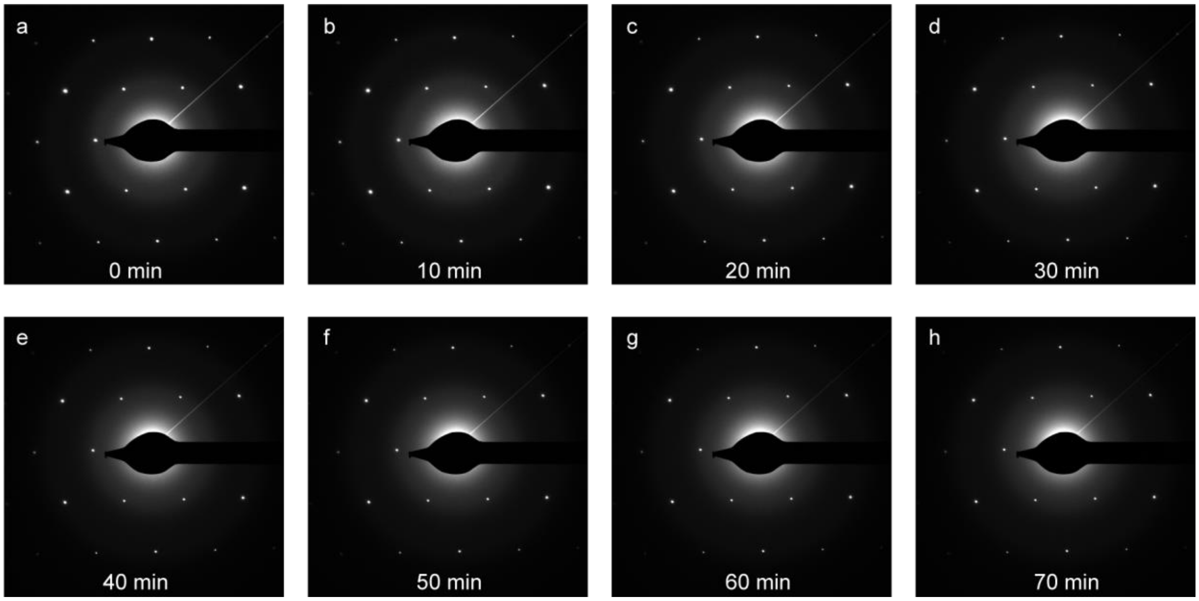

Supplementary Figure 18. The stability of the transfer-free hydrophilic graphene under electron irradiation. The stability of graphene was test by *in-situ* observing the intensity change of selected area electron diffraction (SAED) patterns of graphene in a TEM. **a-h,** SAED patterns from the same position of suspended graphene membrane exposed to electron beam (a dose rate of 204.76 e $Å^{-2}$ s^{-1}) ranging from 0 min to 70 min. The suspended graphene membranes were treated with oxygen plasma for 10 s before the stability investigation. After a long-duration electron irradiation for 70 min, the SAED patterns of the transfer-free graphene were still sharp.

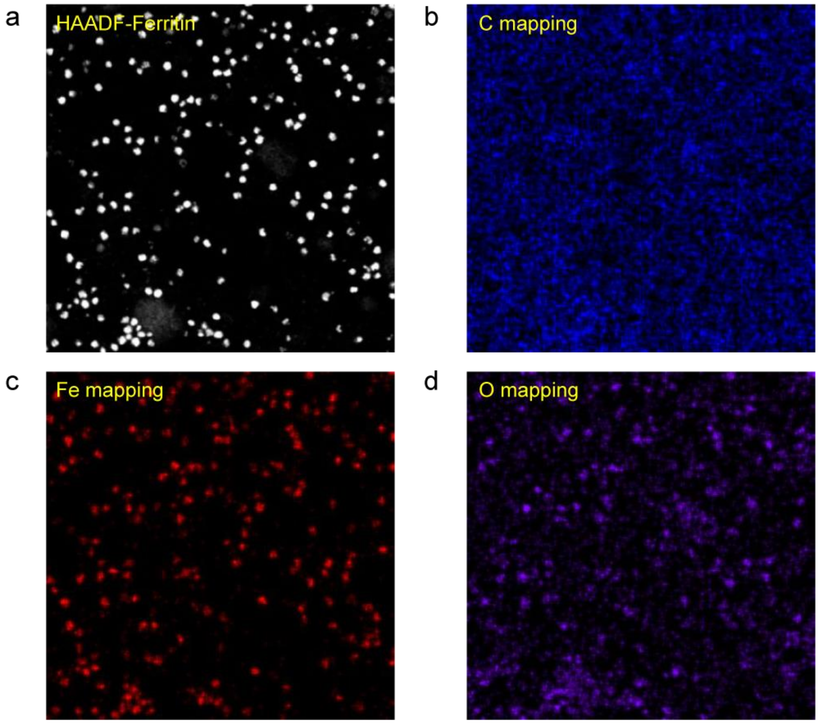

Supplementary Figure 19. Characterizations of ferritins on the graphene membranes. a, HAADF-STEM image of ferritins which dispersed uniformly on the graphene. **b-d,** STEM-EDS map of ferritins. **c,** Fe and O are shown in blue, red and purple, respectively.

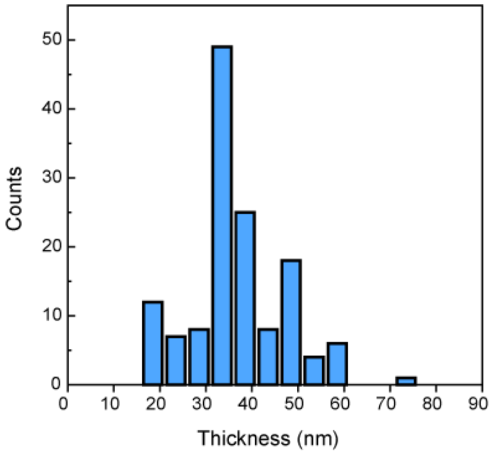

Supplementary Figure 20. Statistics of ice layer thickness from more than 100 locations. The ice thickness measurement was mainly performed at those data collection area that accounted for the majority regions of the grid. Source data are provided as a Source Data file.

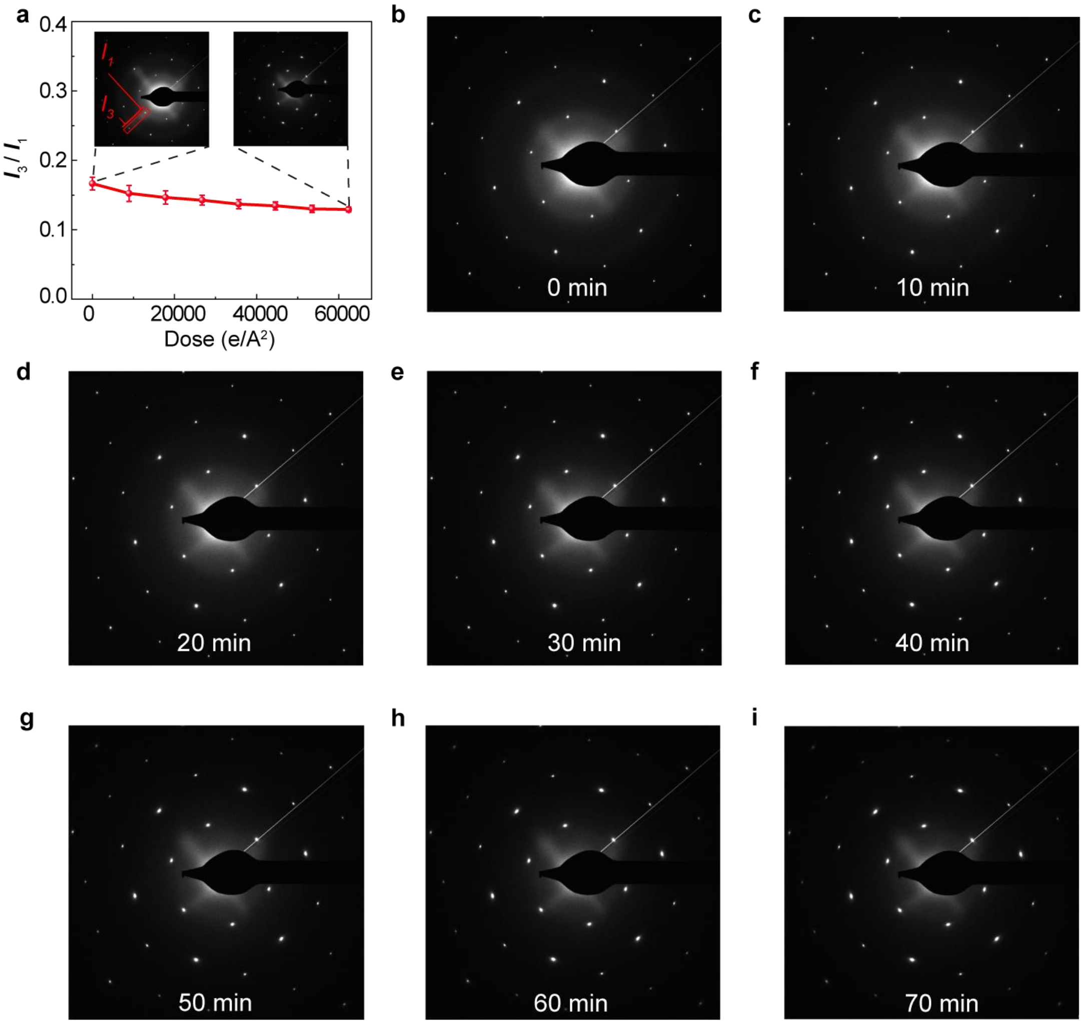

Supplementary Figure 21. Electron beam radiation robustness of graphene grid in cryo-EM. a, Ratio of the third-order integrated Bragg intensity (I_3) to the first-order (I_1) from the SAED patterns of graphene as a function of dose density. Insets: SAED patterns for the same position of the graphene after 1 sec (left) and 70 min (right) of electron-beam exposure (15 e $Å^{-2}$ s^{-1} of dose rate). **b-i,** SAED patterns for the same position of suspended graphene membrane exposed to electron beam ranging from 0 min to 70 min. Source data are provided as a Source Data file.

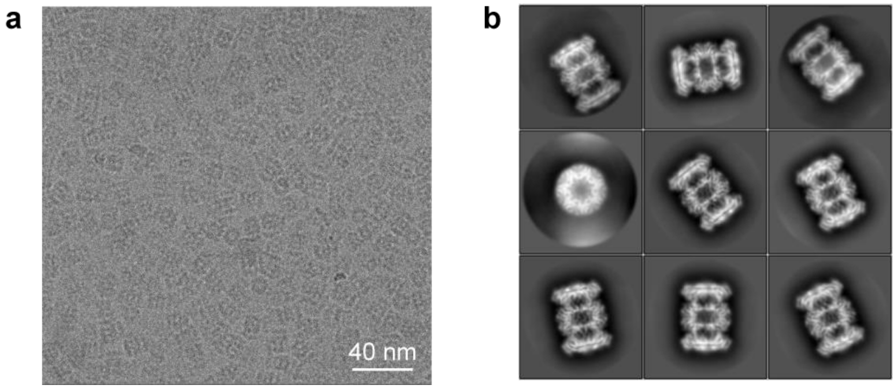

Supplementary Figure 22. Cryo-EM images of 20S proteasome particles on the graphene. a, Distribution of 20S proteasome particles on the graphene. **b,** Representative 2D class averages of the 20S proteasome particles.

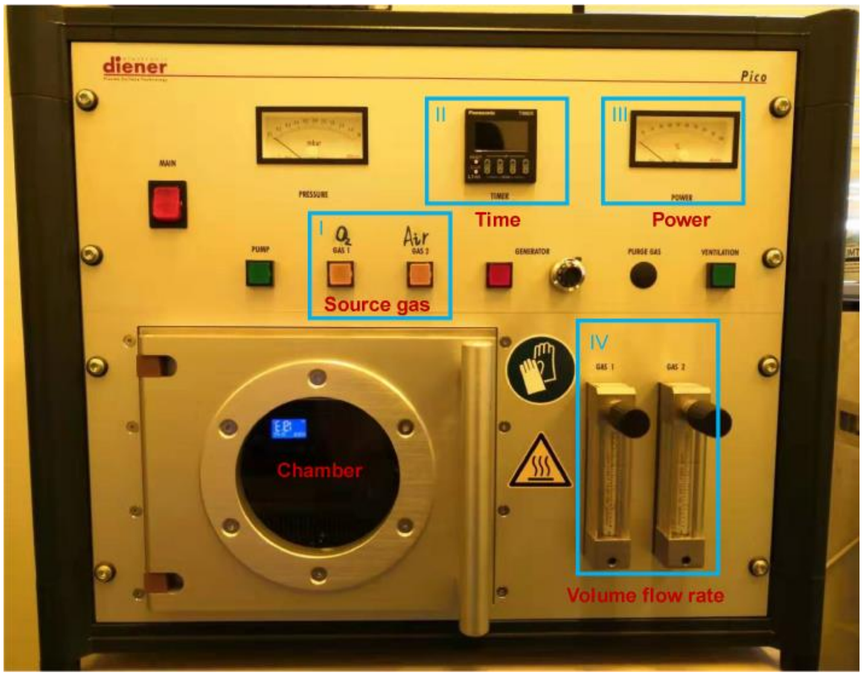

Supplementary Figure 23. Reactive ion etcher. The chamber of reactive ion etcher, the control systems of source gas, volume flow rate, time and power are marked in the optical image, respectively.

Supplementary Table 1. Maximum strain and stresses on suspended graphene membranes during the drying process.

Diameter (μm)	Deflection(max) (μm)	Strain(max) (%)	Stress(max) (GPa)	Liquid
33	0.98	0.23	2.3	water
50	2.92	0.91	9.1	isopropanol

Supplementary Note 1. Radial strain and stress in suspended graphene membrane

The radial strain ε and stress σ in graphene membrane can be estimated as follows. [7]

Supplementary Equation 1 : $\varepsilon = \dfrac{2h^2}{3a^2}$

Supplementary Equation 2: $\sigma = \dfrac{E}{1-\nu}\varepsilon$

Here, *h* is the maximum membrane deflection that can be measured by the digital holographic microscopy (R2200 model, LynceeTec Inc.), and *a* is the initial radius of the membrane. *v* and *E* are the Poisson ratio (~0.165) for graphene and the elastic modulus (~1 TPa)[8], respectively. Therefore, the radial strain ε and corresponding stress σ of suspended graphene can be calculated as shown in Supplementary Table 1.

Both samples were monolayer graphene membranes that ruptured after liquid evaporation. The maximum stresses we recorded are much lower than the theoretical strength of pristine graphene (~120 GPa)[9], corresponding with the previously reported results[10].

Supplementary References

1. Zhang, P. *et al.* Fracture toughness of graphene. *Nat. Commun.* **5**, 3782 (2014).
2. Zandiatashbar, A. *et al.* Effect of defects on the intrinsic strength and stiffness of graphene. *Nat. Commun.* **5**, 3186 (2014).
3. Cancado, L. G. *et al.* Quantifying Defects in Graphene via Raman Spectroscopy at Different Excitation Energies. *Nano Lett.* **11**, 3190-3196 (2011).
4. Araujo, P. T., Terrones, M., Dresselhaus, M. S. Defects and impurities in graphene-like materials. *Mater Today* **15**, 98-109 (2012).
5. Lucchese, M. M., *et al.* Quantifying ion-induced defects and Raman relaxation length in graphene. *Carbon* **48**, 1592-1597 (2010).
6. Childres, I., Jauregui, L. A., Tian, J. F., Chen, Y. P. Effect of oxygen plasma etching on graphene studied using Raman spectroscopy and electronic transport measurements. *New J. Phys.* **13**, 025008 (2011).
7. Small, M. K. & Nix, W. D. Analysis of the Accuracy of the Bulge Test in Determining the Mechanical-Properties of Thin-Films. *J. Mater. Res.* **7**, 1553-1563 (1992).
8. Lee, G. H. *et al.* High-Strength Chemical-Vapor Deposited Graphene and Grain Boundaries. *Science* **340**, 1073-1076 (2013).
9. Xu, J. *et al.* Enhancing the Strength of Graphene by a Denser Grain Boundary. *ACS Nano* **12**, 4529-4535 (2018).
10. Lee, C. K. *et al.* Monatomic Chemical-Vapor-Deposited Graphene Membranes Bridge a Half-Millimeter-Scale Gap. *ACS Nano* **8**, 2336-2344 (2014).
