# Supplementary material for: Robust ultraclean atomically thin membranes for atomic-resolution electron microscopy
Source: Nat Commun. 2020 Jan 28;11:541. doi: 10.1038/s41467-020-14359-0 (PMC6987160; doi:10.1038/s41467-020-14359-0)
Supplement: Supplementary file 3 — Description of Additional Supplementary Files [file 41467_2020_14359_MOESM3_ESM.pdf]

Description of Additional Supplementary Files

File Name: Supplementary Movie 1
Description: **Drying process of monolayer graphene membrane with linear defects.** *In-situ* optical observations showing the crack appeared around the wrinkle of graphene membrane. And the graphene membrane eventually broken when the liquid evaporated.

File Name: Supplementary Movie 2
Description: **Drying process of single-crystal graphene membrane without linear defects.** The single-crystal graphene membrane without wrinkle kept intact during the whole drying process. The white particles on the suspended graphene membrane revealed the existence of graphene.

File Name: Supplementary Movie 3
Description: **Drying process of bilayer graphene membrane.** *In-situ* optical observations revealing the liquid evaporated from the center to the edge of a hole, where the bilayer graphene membrane with wrinkles remains intact after the liquid was totally dried.
